# Effect of Dietary and Physical Activity Interventions Combined with Psychological and Behavioral Strategies on Preventing Metabolic Syndrome in Adolescents with Obesity: A Meta-Analysis of Clinical Trials

**DOI:** 10.3390/nu17132051

**Published:** 2025-06-20

**Authors:** Claudia C. Jiménez-Peláez, Ángel Fernández-Aparicio, Miguel A. Montero-Alonso, Emilio González-Jiménez

**Affiliations:** 1 Hospital de Alta Resolución de Alcaudete, Cam. Viejo de la Fuensanta, 2, 23660 Jaén, Spain; claucrisjipe@hotmail.com; 2 Department of Nursing, Faculty of Health Sciences, University of Granada, 18016 Granada, Spain; emigoji@ugr.es; 3 Instituto de Investigación Biosanitaria (ibs.GRANADA), 18014 Granada, Spain; 4 Department of Statistics and O.I., Faculty of Medicine, University of Granada, 18016 Granada, Spain; mmontero@ugr.es

**Keywords:** adolescents, behavior, diet*, insulin resistance, metabolic syndrome, physical activity, psychology

## Abstract

Bacground/Objectives: Obesity in adolescents is escalating, accompanied by comorbidities such as insulin resistance and cardiovascular disease, which favor the early onset of metabolic syndrome (MetS). There is an urgent need for effective interventions to prevent and treat MetS. We aimed to analyze intervention studies targeting lifestyle improvements in adolescents with obesity. We also determined the effect of combining dietary and/or physical activity interventions with educational, psychological, and emotional support-based interventions compared to traditional interventions in preventing MetS in adolescents with obesity. Methods: The PubMed, Cochrane, Web of Science, CINAHL, and Scopus databases were consulted. Ten clinical trials were included in the systematic review, of which six were eligible for the meta-analysis. Results: Combined interventions significantly decreased body weight (−1.10 [−1.64, −0.55], *p* < 0.001). Although not statistically significant improvements were observed in the meta-analysis for zBMI and waist circumference. The results indicate that diet- and exercise-based interventions are associated with a further decrease in body composition and non-anthropometric variables associated with MetS when combined with educational, psychological, and behavioral interventions. Conclusions: Some studies suggest potential long-term benefits, but further research is necessary to define effective interventions for improving body composition and preventing MetS in obese adolescents, addressing the inconsistencies in current clinical trials. Systematic Review Registration (PROSPERO CRD42023463428).

## 1. Introduction

Obesity, considered a global epidemic by the World Health Organization (WHO), is the chronic non-communicable disease with the highest prevalence worldwide in both adults and adolescents [1]. According to official WHO data, this prevalence has risen dramatically in recent decades, from 4% overweight and obesity among children and adolescents to 18% in 2016 [2]. In this line, it has been observed that the worrying data on obesity prevalence continues with a significant rising tendency after the COVID-19 pandemic [3,4,5]. There is a broad consensus that the main factors predisposing to the development of obesity include the progressive loss of healthy nutritional habits and a decrease in the practice of regular physical activity [3,4]. However, psychosocial and socioeconomic factors, such as stress and low socioeconomic status, have been linked to the development of obesity in different studies [6,7,8].

Given these risk factors, it is crucial to adopt a comprehensive approach to obesity, as metabolic and clinical alterations are appearing among the adolescent population, including insulin resistance and cardiovascular disease at an increasingly early age, both of which favor the early appearance of metabolic syndrome (MetS) [9]. The term MetS describes a series of metabolic disorders or abnormalities that increase the risk of cardiovascular disease and type 2 diabetes mellitus [10,11]. Among these disorders are abdominal obesity, dyslipidemia, arterial hypertension, and abnormalities in glucose metabolism associated with insulin resistance, which is a pathophysiological phenomenon in which the biological action of insulin in the tissues is altered [12,13].

The increasing prevalence of MetS among adolescents has led to numerous intervention studies in which different strategies have been implemented to prevent or treat MetS or any of its components and/or to effect changes in body composition [14]. Traditional interventions typically focus on dietary modifications and physical activity, either independently or in combination [15]. As regards nutritional modification, there is no consensus on the most appropriate type of intervention; some short-term approaches include low-carbohydrate diets or those that are low in sugar and protein-rich and calorie-restricted to a greater or lesser extent [16,17]. In this respect, Mendes et al. [18] showed that an energy reduction of 500 Kcal reduced the body mass index (BMI) and improved insulin and blood pressure (BP) values and the homeostasis model assessment of insulin resistance (HOMA-IR). Other studies, such as that by Krebs et al. [19] on adolescents with obesity, have reported that diets low in carbohydrates and rich in protein can rapidly improve the lipid profile. However, it has also been observed that in order to achieve long-term improvement, any dietary intervention must be complemented with regular physical exercise [17]. The combination of diet and aerobic physical exercise seems to promote body weight (BW) control and improve the lipid profile and general health status [20]. Other intervention studies based on the implementation of strength-and-resistance training, together with an energy restriction of 250 Kcal, have proven their effectiveness in the adolescent population, achieving significant reductions in BMI, body fat (BF) percentage, waist circumference (WC), and an improved lipid profile [21,22].

Despite the benefits of dietary and exercise-based interventions, their long-term adherence remains a major challenge. In response, researchers have increasingly emphasized the importance of psychological and behavioral interventions to enhance compliance and maximize health outcomes [23,24]. Thus, Wu et al. [25] and Bean et al. [26], in their respective intervention studies, observed that a combined treatment approach based on diet, physical exercise, and motivational interviews achieved better control of body weight and reduced caloric intake in adolescents with obesity. Similar results have been described by Walpole et al. [27] and Gourlan et al. [28], who emphasized the importance of motivational interviewing as a means of encouraging adolescents with obesity to achieve better control of their body weight.

Treatment approaches for adolescents with obesity can be individual or wide-ranging, encompassing family and school environments [29,30]. In this sense, Ranucci et al. [31] developed a multicomponent intervention that combined nutritional monitoring, physical exercise, and psychological care with a family-based approach. The participants in this study achieved significant reductions in BMI and waist circumference and increases in fat-free mass, demonstrating the efficacy of the intervention.

In recent years, increasing attention has been paid to the psychological and environmental factors that may affect eating patterns, as well as the long-term limitations of purely dietary and exercise-based approaches [32,33,34]. Adolescents are more vulnerable to environmental factors since they are in the midst of pubertal development, personal growth, and the search for their personal identity [35]. This has led to a growing interest in educational, psychological, and emotional support-based interventions aimed at improving weight management and metabolic health. However, there is currently no consensus on the optimal combination of interventions for managing obesity in adolescents and preventing MetS.

Although previous systematic reviews have addressed the efficacy of weight-loss interventions, including psychological components, in school-age participants with obesity [36,37], to our knowledge, there are currently no published systematic reviews or meta-analyses that have specifically addressed the effectiveness of dietary and physical interventions combined with psychological interventions in the prevention of MetS in adolescents with obesity. Therefore, the aim of the present systematic review was to examine the characteristics and describe the outcomes achieved after carrying out intervention studies focused on improving lifestyle habits, especially dietary and physical activity patterns, in adolescents with obesity. In addition, the effect of dietary and/or physical activity interventions when combined with educational, psychological, and emotional support-based interventions for MetS prevention in adolescents with obesity was determined.

## 2. Materials and Methods

### 2.1. Databases and Search Strategy

This systematic review and meta-analysis were reported in accordance with the PRISMA recommendations [38] of 2020, and its protocol was published in PROSPERO (reference CRD42023463428). The purpose of the present work was to identify clinical trials conducted to evaluate the effects of interventions such as lifestyle modification and emotional support on MetS and its components in adolescents with obesity. To do so, a bibliographic search was carried out in the Web of Science, CINAHL, Cochrane, and Scopus databases and with the PubMed search engine. A time filter was applied both in the database searches and to the application of the search engine to restrict results to the period January 2008–November 2023. An advanced search was carried out in PubMed, selecting the following options: type of article “clinical trial” and “randomized controlled trial”, species “humans”, language “English”, and age “child-6–12 years” and “adolescent-13–18 years”. In CINAHL, an advanced search was carried out, with the following options: language limiters “English”, type of article “clinical trial” and “randomized controlled trial”, and age “child 6–12 years” and “adolescents 13–18 years”. In Scopus, a basic search was carried out, refined by the type of document “article” and language “English”. In Cochrane, an advanced search was performed, refined by publication type “clinical trials”. In the Web of Science, a basic search was used, refined by document type “clinical trial” and language “English”.

The terms used in the search strategy were based on the following Medical Subject Headings (MeSH) descriptors: insulin resistance, metabolic syndrome, diet*, teens, adolescents, and physical activity. The search strategy used in all databases was: “metabolic syndrome” AND “insulin resistance” AND teens OR adolescents AND diet* AND “physical activity”.

### 2.2. Selection of Studies/Eligibility Criteria

Studies were selected for this review in two phases. In the first phase, the titles and abstracts were reviewed to select potentially relevant studies (Á.F.-A and C.C.J.-P), according to the following inclusion criteria: (1) randomized and non-randomized clinical trials; (2) adolescents aged 10–19 years according to the range established by the WHO [39]; (3) adolescents identified with obesity according to cut-off points or obese classifications specific for the adolescent population; (4) studies carrying out dietary and/or physical activity interventions combined with educational, psychological, and emotional support-based interventions; (5) articles published in English; (6) articles published during the period 2008–23; and (7) access to the full text. In case of doubt, the full text of the article was analyzed further. In the second phase, the eligibility of the articles was determined (Á.F.-A and C.C.J.-P) by analyzing the full text and excluding the following: (1) studies in which no analysis was performed of anthropometric parameters; (2) studies including overweight population, or those where the inclusion of adolescents with obesity is unclear; (3) studies including adolescents with obesity suffering from type 2 diabetes mellitus, arterial hypertension, or dyslipidemias, or diagnosed with metabolic syndrome; (4) studies including adolescents with obesity suffering from obesity-related complications or another type of comorbidities; (5) observational studies, secondary studies, and intervention studies with no control groups. This phase of study selection was carried out by two reviewers, and in case of doubt, a third reviewer (E.G.-J.) was consulted.

### 2.3. Data Extraction

Once the articles to be included in the review were chosen, relevant data were extracted and grouped into five categories: authors and year, study information (sample size, number of participants per group, age of participants, population, and diagnostic criteria for inclusion), characteristics of the intervention, and study duration. Regarding outcome measures, we extracted the following values: BW, BMI, BF, WC, fasting blood glucose (FBG), systolic blood pressure (SBP), diastolic blood pressure (DBP), HDL, triglycerides (TG), HOMA-IR, and insulin sensitivity index (ISI). Data extraction was performed by two reviewers (Á.F.-A and C.C.J.-P), and in case of doubt, a third reviewer (E.G.-J.) was consulted. 

### 2.4. Assessment of Methodological Quality and the Risk of Bias

The methodological quality and risk of bias of the randomized controlled trials included in the review were determined using the Cochrane risk-of-bias tool for randomized trials (ROB 2) [40] and the Jadad scale [41]. The Risk of Bias in Non-randomized Studies-of Interventions (ROBINS-I) tool was used to assess the methodological quality and risk of bias of the non-randomized controlled trials included in the review.

The ROB 2 tool assesses different types of biases through the following six domains: randomization process, deviations from intended interventions, missing outcome data, measurement of the outcome, selection of the reported result, and overall bias. Within each of these domains, various questions are considered, and according to the responses obtained, studies may be classed as “Low risk”, “High risk” or “Some concerns” (if there is insufficient information to adequately assess the risk of bias) [40].

The Jadad scale was used to assess the methodological quality and risk of bias of the randomized controlled trials considered. This scale assesses the risk of bias in three domains: randomization bias (0–2 points), blinding bias (0–2 points), and attrition bias (0–1 points). Thus, the potential score for bias ranges from 0 to 5 points. A score of 0–2 on the scale indicates low quality, and one of 3 or more indicates high quality. Both the Jadad scale and Cochrane tool were applied by two reviewers (Á.F.-A and M.A.M.-A). In case of doubt, a third reviewer (E.G.-J.) was consulted.

The ROBINS-I tool was also applied by two reviewers (Á.F.-A and M.A.M.-A), and in doubtful cases, a third reviewer (E.G.-J.) was consulted. This tool assesses seven domains of bias: confounding and selection bias before the start of the trial, bias in the classification of interventions, and the remaining four domains assess biases due to deviations from intended interventions, missing data, measurement of outcomes, and selection of the reported result. According to the responses obtained, studies may be classified as “Low risk”, “Moderate risk”, “Serious risk”, and “Critical risk” of bias [42].

### 2.5. Synthesis of Results/Data Analysis

Data extracted for each outcome measure were transferred to Microsoft Excel (version 16.0.10406.20006, Microsoft Corporation Inc., Redmond, Washington). A meta-analysis was carried out using R 4.3.2 software [43] and Jamovi (version 2.4.) [44]. Statistical significance was set at *p* < 0.05. A random-effects model was used to assess the standardized mean differences (SMD) and their 95% confidence intervals, which were determined as the outcome measures for BW, WC, and zBMI. Studies included in the meta-analysis were those that compared groups receiving dietary and/or physical activity interventions with groups that received the same interventions combined with educational, psychological, and emotional support-based components. Additionally, the meta-analysis considered differences in the units of measurement used across studies (e.g., mmol/L, mg/dL) and how data were reported, whether as means with ranges, standard deviations (SD), or standard errors (SE). These factors affected the number of studies included in each outcome-specific meta-analysis.

The SMD represents the magnitude of the intervention effect in each study relative to the observed variability within that study, or in other words, corresponds to the effect size known in social sciences as Hedges’ (adjusted) g [45]. In our study, SMDs were calculated by comparing the pre-and post-intervention changes between the experimental and control groups. This approach corresponds to a difference-in-differences (DiD) analysis that adjusts for baseline differences and time trends. The SMD was calculated as follows:

SMD = [M_Post,IG_ − M_Pre,IG_] − [M_Post,CG_ − M_Pre,CG_]/Standard deviation of outcome among participants

where:

M_Post,IG_ = Mean of the intervention group after the intervention.

M_Pre,IG_ = Mean of the intervention group before the intervention.

M_Post,CG_ = Mean of the control group after the intervention.

M_Pre,CG_ = Mean value of the control group before the intervention.

In the random-effects model, the control group included dietary interventions, physical interventions, or both, while the intervention group included the previous interventions combined with educational, psychological, and emotional support-based interventions). Heterogeneity was evaluated by estimating the restricted maximum-likelihood estimator (tau^2^) [46], I^2^ statistic, and Q-test for heterogeneity [47]. The interpretation of the I^2^ statistic is as follows: I^2^ < 25%: low; I^2^ 25–50%: moderate; I^2^ > 50%: high [48]. In the case of tau^2^ > 0, regardless of the results of the Q-test, a prediction interval for the true outcomes was obtained.

Funnel plots were not obtained because they are not recommended to be conducted in meta-analyses, including fewer than 10 studies [49].

## 3. Results

### 3.1. Study Selection

**Figure 1:**
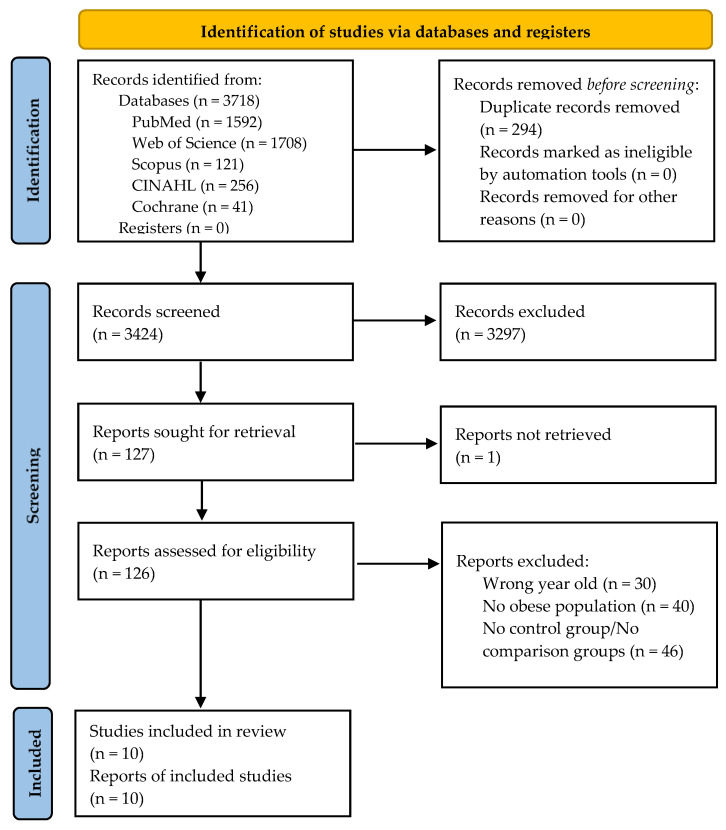
Flow diagram of the study selection process.

The search strategy, carried out in the different databases and with the PubMed search engine, with a filter restricting studies to those published between January 2008 and November 2023, produced 3718 results: 1708 were from Web of Science, 41 from Cochrane, 1592 from PubMed, 256 from CINAHL, and 121 from Scopus. After eliminating duplicate publications, the titles and abstracts of 3424 articles remained for review. After applying the inclusion and exclusion criteria, 127 articles were sought for retrieval, and one was not found [50]. The full texts of 126 articles were analyzed to determine their eligibility. Finally, 10 articles were chosen for inclusion in the systematic review, of which six were included in the meta-analysis. A reverse literature search of the selected articles was not conducted. Figure 1 shows a flow diagram of the study selection and exclusion procedure applied in accordance with the PRISMA recommendations [38].

### 3.2. Characteristics of the Studies Included

Table 1 summarizes the characteristics of the clinical trials included in this review. Three studies were carried out in the USA. The remainder were conducted in Brazil (n = 2), Iran (n = 1), Australia (n = 1), Indonesia (n = 1), Serbia (n = 1), and Germany (n = 1). The largest sample size was 474, and the smallest sample size was 22. The follow-up period of the studies ranged from 4 to 52 weeks. Of the included studies, three were non-RCTs, and seven were RCTs. Six studies included both girls and boys, three included only girls, and one did not specify the sex of the participants.

### 3.3. Effects of Interventions on Body Composition and Variables Associated with MetS

Table 2 shows the changes in body composition and MetS parameters after the interventions. Two studies included in the review involved dietary interventions combined with educational sessions [51,52]. Truby et al. [51] applied a 20% energy reduction to the intervention group, which was given a structured low-fat diet for 12 weeks together with a program of dietary advice prior to the study; the control group consumed a normal diet for the same period. By the end of the intervention, participants in the intervention group presented significant decreases in BMI, BW, BF, WC, and HOMA-IR. In a study by Normayanti et al. [52] on adolescents with obesity in Indonesia, a nutritional education booklet on the Dietary Approaches to Stop Hypertension diet was applied once weekly for four weeks, achieving a significant decrease in BMI, WC, and SBP [52].

Similarly, Plavsic et al. [53] conducted a study based on interventions that combined physical exercise and dietary interventions based on hypocaloric diets, along with educational sessions. By the end of the interventions, the authors reported significant reductions in BW, BMI, BF, and WC [53]. In addition, an improvement in systolic and diastolic BP and sensitivity to insulin has been reported [53].

The interventions described in seven of the studies included in our review involved multiple components: exercise, diet, health education sessions, and cognitive behavioral therapy [54,55,56,57,58,59,60]. The main findings reported by all these studies were a greater reduction in the intervention groups in BW [54], BMI [54,55], BF [54,55,57,58], WC [55,60], and an improvement in blood pressure levels [55,60]. Regarding the therapies focused on health educational sessions, therapies, or behavior-modification therapies, all of the studies mentioned above applied them to participants in joint sessions with parents, except for the study performed by Ackel-D’Elia et al. [54]. In the latter study, parental participation consisted of recording dietary intake with their children.

### 3.4. Random-Effect Meta-Analyses of Changes in Body Weight, Waist Circumference, and zBMI by Combined Interventions

**Figure 2:**
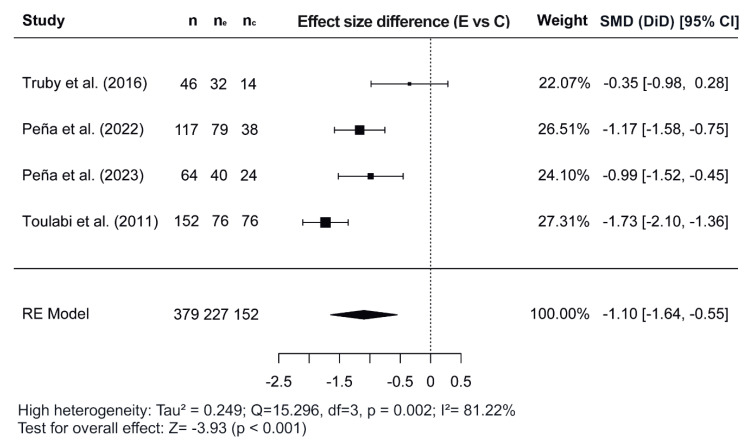
Forest plot showing the standardized mean differences (SMD) for changes in body weight based on a difference-in-differences (DiD) analysis. Each SMD represents the difference in weight change from baseline to follow-up between the experimental (combined interventions) and control (traditional interventions) groups [51,56,57,58]. E vs. C, Experimental group compared to Control group; n, total study sample size; n_e_, sample size of experimental group; n_*c*_, sample size of control group; SMD, standardized mean difference; CI, confidence interval; RE, random effects model.

**Figure 3:**
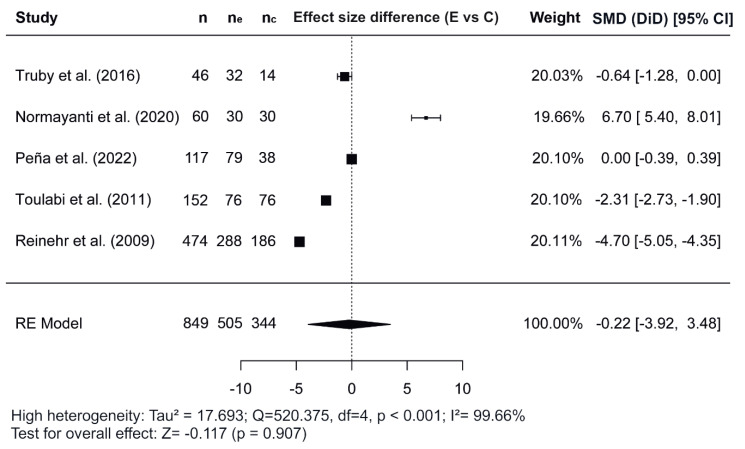
Forest plot showing the standardized mean differences (SMD) for changes in waist circumference based on a difference-in-differences (DiD) analysis. Each SMD represents the difference in waist circumference from baseline to follow-up between the experimental (combined interventions) and control (traditional interventions) groups [51,52,56,58,60]. E vs. C, Experimental group compared to Control group; n, total study sample size; n_e_, sample size of experimental group; n_*c*_, sample size of control group; SMD, standardized mean difference; CI, confidence interval; RE, random effects model.

**Figure 4:**
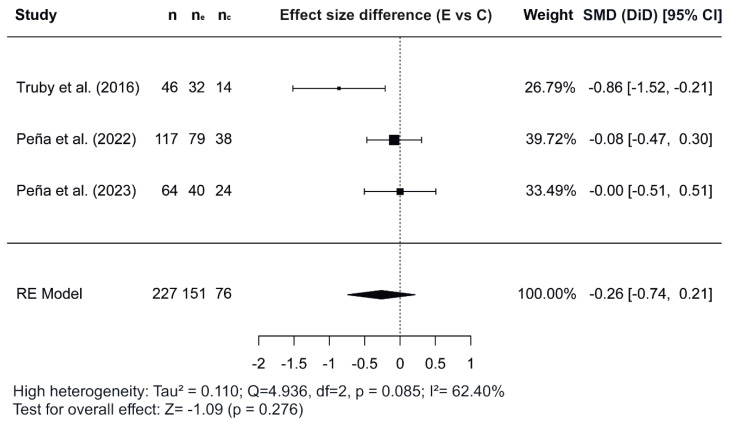
Forest plot showing the standardized mean differences (SMD) for changes in zBMI based on a difference-in-differences (DiD) analysis. Each SMD represents the difference in zBMI from baseline to follow-up between the experimental (combined interventions) and control (traditional intervention) groups [51,57,58]. E vs. C, Experimental group compared to Control group; n, total study sample size; n_e_, sample size of experimental group; n_*c*_, sample size of control group; SMD, standardized mean difference; CI, confidence interval; RE, random effects model.

Figure 2, Figure 3 and Figure 4 show the forest plots of the effects of the combined interventions on BW, WC, and zBMI, respectively, in comparison to the control groups. The effects of the combined interventions were analyzed in four studies for BW [51,56,57,58], five studies for WC [51,52,56,58,60], and three studies for zBMI [51,57,58]. Compared with the control groups, the experimental groups showed a significantly greater reduction in BW (SMD for DiD = −1.10; 95% CI = −1.64, −0.55; z = −3.93; *p* < 0.001) from baseline to post-intervention. However, there was no significant decrease for WC (SMD = −0.22; 95% CI = −3.92, 3.48; z = −0.117; *p* = 0.907) and for zBMI (SMD = −0.26; 95% CI = −0.74, 0.21; z = −1.09; *p* = 0.276).

### 3.5. Evaluation of the Methodological Quality

The risk of bias in each clinical trial included in a systematic review must be determined by assessing its internal validity. In the present review, this was done using the revised Cochrane risk-of-bias tool for randomized trials (ROB 2), which includes a “risk of bias” table in which each item is assessed, thus providing a final evaluation that determines the quality of the studies included [40]. One of the most commonly used scales for assessing the quality of RCTs is the Jadad scale [41]. The risk of bias of the NRCTs included in this work was evaluated using the ROBIN-I tool [42]. Table 3 and Table 4 present the results obtained by the RCTs and NRCTs included in this review, respectively.

## 4. Discussion

In this systematic review, we considered not only the characteristics of the various intervention strategies for preventing metabolic syndrome implemented in programs for adolescents with obesity but also the outcomes of these programs in terms of the participant’s body composition parameters. In general, each of the intervention strategies employed had a positive impact on the body composition parameters considered and alleviated MetS components. In addition, a meta-analysis was conducted to determine the effect of combining exercise and dietary interventions with educational, psychological, and emotional support-based interventions.

According to the studies included in this review, different dietary programs produced greater improvements in obesity-related anthropometric parameters when participants received nutritional education sessions [51,52]. Moreover, Truby et al. [51] reported a significant reduction in HOMA-IR, and Normayanti et al. [52] recorded a decrease in SBP values. Given the similar benefits reported by these authors, it could be suggested that combining dietary programs with nutritional counseling from dietitians could be more effective in improving obesity status and associated insulin resistance. Supporting this conclusion, Gow et al. [61] performed a systematic review of the effects of nutritional interventions with different macronutrient compositions on BMI and reported similar improvements in body weight values, regardless of macronutrient distribution. Furthermore, one of the studies included in our review, conducted by Plavsic et al. [53], implemented a physical exercise program with energy restriction and reported optimal results in anthropometric measurements, particularly among participants who also received nutrition education sessions. This study also observed statistically significant changes in both systolic and diastolic blood pressure levels. Although the findings are not entirely conclusive, the combination of physical exercise and energy-restricted diets seems to improve anthropometric parameters in adolescents with obesity and those associated with MetS, especially when complemented by dietary counselling sessions.

In line with the potential advantages of combining traditional interventions with nutrition education sessions, a systematic review conducted by Aguilar-Cordero et al. [62] concluded that implementing multicomponent interventions is effective in promoting lifestyle changes and facilitating the acquisition of new, healthier habits. In the studies by Ackel-D’Elia et al. [54] and Bianchini et al. [55], in which a multicomponent intervention was implemented, BMI values fell significantly, and reductions in the percentage of body fat and increases in fat-free mass were observed. These results are consistent with those described by Shrewsbury et al. [63], who studied a group of Australian adolescents using an intervention based on lifestyle modification and resistance exercises. In this case, the intervention group achieved reductions in BMI, waist circumference, and cholesterol levels. However, two studies conducted by Peña et al. [57,58] reported significant decreases in body fat percentage, but no substantial changes in BMI were observed in adolescents with obesity. Of interest in this context is the fact that participants with obesity in these two studies were also diagnosed with prediabetes.

The family-based multicomponent interventions described by Bianchini et al. [55] and Reinehr et al. [60] achieved improvements in the WC of participants and significant reductions in systolic and diastolic blood pressure. These results are partially consistent with those presented by Savoye et al. [64] in their study conducted on American adolescents, who achieved improvements in BMI, body fat, lipid profile, and HOMA-IR index. According to Toulabi et al. [56], the implementation of a school-based multicomponent intervention improved BMI, BW, and WC values. These findings coincide with those reported by Nayak et al. [65], who conducted a study with 194 adolescents from South India; these participants achieved lower BMI values after taking part in a multi-component intervention based on lifestyle modifications and increased aerobic physical exercise at school.

Regarding the meta-analysis, SMD was obtained and analyzed to determine the effects of combined interventions on body composition parameters compared to the control groups. Significant effects were observed for BW in combined interventions [51,56,57,58]. However, non-significant effects were observed for WC and zBMI. In the variable WC, of the five studies meta-analyzed [51,52,56,58,60], the study conducted by Normayanti et al. [52] reported a favoring control group. Of interest in this context is the fact that this study included only girls as participants. Regarding findings obtained in the variable zBMI, the two studies carried out by Peña et al. [57,58] did not report any changes, probably due to the fact that their participants had been diagnosed with prediabetes at baseline.

The present study shows that dietary and exercise-based interventions are associated with a further decrease in body composition and MetS-associated parameters in adolescents with obesity and may have longer-lasting effects when combined with educational and psychological interventions. However, this tendency is only partially supported by the results of the meta-analysis, which showed a low effect size, probably due to the heterogeneity of the existing and included studies and also by their specific nature or the duration of follow-up. Therefore, based on the findings of this review, it would be advisable to continue conducting studies on adolescents with obesity, addressing dietary, physical, psychosocial, and educational dimensions in an integrated manner to standardize and achieve more robust and effective results.

The systematic review and meta-analysis presented in this study have both strengths and limitations. Its main strength is the novel approach adopted, in that a detailed analysis is made of the characteristics presented by each of the intervention studies considered and of the effects produced on MetS and body composition in the adolescent populations addressed. To this end, the 2020 PRISMA recommendations, widely recognized as valid for structuring systematic reviews of randomized clinical trials and for assessing the interventions performed, were followed [38]. Another strength of the study is that the ROB 2 tool and the Jadad scale were used to assess the risk of bias of the studies included in the review; the latter scale was also used to assess the methodological quality of these studies. The ROBINS-I tool was used to assess the methodological quality and risk of bias of the non-randomized controlled trials. In order to lessen the risk of subjectivity; the initial study selection, data extraction, and evaluation of the studies were performed by two independent reviewers, who consulted a third reviewer if any doubt arose.

The large number of databases included in this review is another strength. However, a limitation is the fact that only databases from periodical publications were consulted, while specific databases of clinical trials or other sources of data from unpublished materials were excluded, which may have given rise to some selection bias [66]. Another limitation is that publication bias could not be fully evaluated because funnel plots and Egger’s test were not reported, as they are only recommended to be conducted when each outcome measure includes more than 10 studies [49]. Finally, the clinical trials included in this meta-analysis presented a certain degree of heterogeneity; therefore, the results should be interpreted with caution.

## 5. Conclusions

Scientific evidence corroborates the importance of intervention strategies focused on reducing obesity among adolescents as an essential element for preventing MetS and promoting positive changes in body composition. Dietary intervention studies have documented improvements in this respect, regardless of the type of diet prescribed, as long as the caloric intake is reduced. However, a significant reduction in body fat percentage and BMI values has been observed in intervention studies based on diet and aerobic and resistance forms of physical exercise. The changes thus obtained are greater and tend to be longer-lasting when cognitive behavioral therapy and educational intervention are simultaneously applied to dietary and physical exercise interventions. Despite these observations, the lack of consensus on many questions and the significant variability in the interventions considered in this review highlights that the currently available interventions in adolescents are far from the target of controlling obesity in adolescents. Therefore, further analysis is needed to define the characteristics required for an intervention aimed at improving body composition and preventing the development of MetS in adolescents.

## Figures and Tables

**Table 1:** Characteristics of the studies included in the systematic review.

Study ID		Population								Intervention	Outcomes Measured
Author(s)/Date	Study Design	Country	Initial Sample Size	Final Sample Size	Loss of Follow-Up	Follow-Up	Sex	Age (Ranges)	Inclusion Criteria	Groups	
Truby et al. [51]	RCT	Australia	n = 50 IG: 36 CG: 14	n = 46 IG: 32 CG: 14	8%	12 weeks	♂ (28%) ♀ (72%)	10–19	BMI > 90th percentile, as defined by CDC 2000	Dietary plus education intervention IG: five sessions of in-person counseling with participants and caregivers plus two sessions by phone. Structured low-fat diet” (55% carbohydrate, 20% protein, 25% fat). CG: Without dietary advice during the study. At the end of the study, participants received the dietary program of the IG.	BMI, BMI z-score, BW, BF (%), WC, HDL, TG, HOMA-IR
Normayanti et al. [52]	non-RCT	Indonesia	n = 60 IG:30 CG:30	n = 60 IG:30 CG:30	0%	4 weeks	♀ (100%)	14–17	BMI ≥ 95th percentile (or ≥26.3 kg/m^2^)	Dietary plus education intervention. IG: Given a nutritional education booklet on dietary approaches to stop hypertension (DASH)), discussed orally for ± 20 min (once weekly for 4 weeks) CG: No nutritional education was given.	BMI, WC, SBP, DBP
Plavsic et al. [53]	RCT	Serbia	n = 44 IG: 22 CG: 22	n = 39 IG: 19 CG: 20	11.37%	12 weeks	♀ (100%)	Range not defined (15.8 ± 1.6)	(BMI) = 33.0 ± 3.1 kg/m^2^	Multicomponent intervention IG: Hypocaloric diet (1500–1700 kcal/day) and 4–6 individual sessions of dietary advice + HIIT training 2 days a week. CG: Hypocaloric diet (1500–1700 kcal/day) and 4–6 individual sessions of dietary advice. Participants of all groups received individual sessions of dietary advice	BW, BMI, BF, WC, SBP, DBP, TG, HDL, HOMA-IR, ISI
Ackel-D’Elia et al. [54]	RCT	Brazil	n = 48 IG: 24 CG: 24	NR	NR	6 months	♂ (33.33%) ♀ (77.77%)	15–19	BMI > 95th percentile (CDC 2000)	Multicomponent intervention IG: Aerobic plus resistance training (180 min/week). CG: Aerobic training (180 min/week) All participants received individual nutritional counseling, psychological therapy for 1 h a week, and a session with an endocrinologist once a month.	BW, BMI, BF, FBG, HOMA-IR
Bianchini et al. [55]	non-RCT	Brazil	n = 97 IG: 50 CG: 47	n = 86 IG:44 CG:42	11.34%	16 weeks	♂ (44.19%) ♀ (55.81%)	10–18	Obesity according to criteria indicated by Cole et al.	Multicomponent intervention. IG: 1 h per week of CBT, 1 h of nutritional intervention, 3 h per week of physical exercise, and once a month, meetings with the professionals and parents CG: No intervention. Just monitoring.	BW, BMI, BF, WC, SBP, DBP, HDL, TG, FBG, HOMA-IR
Toulabi et al. [56]	RCT	Iran	n = 152 IG: 76 CG: 76	NR	NR	6 months	NR	14–19	BMI ≥ 28 (15 years); BMI ≥ 29 (16–17 years)	Behavior-modification intervention. School-based intervention IG: 8 sessions of 45 min of nutritional instructions and techniques to increase physical activity, plus exercise, 1 h per day, 3 days a week, for 6 weeks. CG: Educational brochures were provided after data collection.	BW, BMI, WC
Peña et al. [57]	RCT	USA	n = 64 IG: 40 CG: 24	n = 64 IG: 40 CG: 24	0%	6 months	♂ (60.9%) ♀ (39.1%)	12–16	Latino adolescents, BMI ≥ 95th percentile for age and sex, and prediabetes diagnosed.	Multicomponent intervention IG: 1 day/week (75 min per session) of nutrition and health education with behavior change skills training and 3 days/week (60 min per session) of physical activity. CG: Usual care on T2DM risk factors.	BW, BMI, BMI z-score, BF, ISI
Peña et al. [58]	RCT	USA	n = 117 IG: 79 CG: 38	n = 108 IG: 71 CG: 37	7.70%	6 months	♂ (61.40%) ♀ (38.60%)	12–16	Latino adolescents, BMI ≥ 95th percentile for age and sex, and prediabetes diagnosed.	Multicomponent intervention IG: 1 day/week (75 min per session) of nutrition and health education with behavior change skills training and 3 days/week (60 min per session) of physical activity. CG: Usual care on T2DM risk factors.	BW, BMI, BMI z-score, WC, BF (%), ISI, FBG,
Kitzman-Ulrich et al. [59]	RCT	USA	n = 22 IG 1: 14 IG 2: 13 CG: 8	n = 22 IG 1: 14 IG 2: 13 CG: 8	0%	16 weeks	♀ (100%)	12–15	BMI ≥ 95th percentile.	Familiar therapy IG 1: Multifamiliar therapy + psychoeducation. IG 2: Psychoeducation CG: Waiting list.	↓ non-significant BMI z score only in the psychoeducation group
Reinehr et al. [60]	non-RCT	Germany	n= 474 IG: 288 CG: 186	n= 474 IG: 288 CG: 186	0%	12 months	♂ (43.88%) ♀ (56.12%)	10–16	Adolescents with obesity without endocrine disorders, familial hyperlipidemia or syndromal obesity.	Multicomponent and lifestyle intervention IG: physical activity (aerobic training once a week), nutrition education (twice a month), behavior therapy (twice a month), and psychological family therapy (30 min/month). CG: 15 min of nutritional counseling, physical activity, and behavior patterns.	WC, FBG, HDL, TG, SBP, DBP

*Note.* WHO: World Health Organization; BF: Body fat; BMI: Body mass index; BW: Body weight; CBT: Cognitive behavioral therapy; CDC: Centers for Disease Control and Prevention; CG: Control group; DBP: Diastolic blood pressure; FBG: Fasting blood glucose; HDL: High-density lipoprotein; HOMA-IR: Homeostatic Model Assessment for Insulin Resistance; IG: Intervention group; ISI: Insulin sensitivity indices; MD: Mean differences; NR: Non-reported; RCT: Randomized controlled trial; SBP: Systolic blood pressure; TG: Triglycerides; WC: Waist circumference; ♀: girls; ♂: boys; ↓: reduction.

**Table 2:** Description of the changes achieved in body composition and other variables associated with MetS (glycemia, dyslipidemia, and blood pressure levels) after carrying out intervention studies focused on improving eating and physical activity patterns in adolescents with obesity.

Author(s)/Date	Variables	IG			CG			ΔDiD (ΔIG–ΔCG)
		Pre	Post	Δ	Pre	Post	Δ	
Truby et al. [51]	BW (kg)	86.63 ± 22.60	86.26 ± 23.38	–0.37 ^†,^*	94.42 ± 30.94	96.88 ± 31.00	2.46 ^†^	−4.15
	BMI (kg/m^2^)	32.62 ± 5.9	31.88 ± 6.17	–0.74 ^†,^*	35.17 ± 8.54	35.74 ± 8.66	0.57 ^†^	−1.58
	BMI z-score	2.19 ± 0.39	2.10 ± 0.46	–0.09 ^†,^*	2.27 ± 0.43	2.29 ± 0.42	0.02 ^†^	−0.13
	BF (%)	39.67 ± 6.38	39.54 ± 5.22	–0.13 ^†,^*	40.36 ± 5.31	42.98 ± 4.26	2.62 ^†^	−2.99
	WC (cm)	105.30 ± 13.53	102.88 ± 14.96	–2.42 ^†,^*	112.44 ± 19.27	113.26 ± 19.54	0.82 ^†^	−3.07
	HDL (mmol/L)	1.1 ± 0.2	1.0 ± 0.2	–0.1 ^†^	1.0 ± 0.3	1.1 ± 0.4	0.1 ^†^	−0.1
	TG (mmol/L)	1.2 ±0.5	1.2 ± 0.4	0.0 ^†^	1.3 ± 0.5	1.2 ± 0.7	–0.1 ^†^	0.1
	HOMA-IR	1.7 ± 1.0	1.5 ± 0.9	–0.2 ^†,^*	2.5 ± 2.1	2.7 ± 1.1	0.2 ^†^	−0.7
Normayanti et al. [52]	BMI (kg/m^2^)	30.68 ± 1.50	30.31 ± 1.50 ^a^	–0.36 ± 0.05 *	30.54 ± 1.60	30.83 ± 1.51 ^a^	0.29 ± 0.05	–0.65 ^†^
	WC (cm)	89.63 ± 7.68	88.79 ± 7.69 ^a^	–0.84 ± 0.14 *	89.55 ± 6.00	90.29 ± 6.16 ^a^	0.75 ± 0.30	–1.59 ^†^
	SBP (mmHg)	113.67 ± 5.15	112.53 ± 5.11 ^a^	–1.13 ± 1.36 *	112.40 ± 5.81	112.93 ± 5.86	0.53 ± 1.81	–1.66 ^†^
	DBP (mmHg)	72.40 ± 5.26	72.70 ± 6.37	−0.13 ± 3.06	72.80 ± 5.96	73.13 ± 7.40	0.33 ± 3.02	–0.46 ^†^
Plavsic et al. [53]	BW (kg)	90.1 ± 11.8	85.4 ± 11.8 ^b^	–4.7 ^†^	89.3 ± 13.8	87.1 ± 15.0 ^c^	–2.2 ^†^	–2.5 ^†^
	BMI (kg/m^2^)	32.6 ± 2.7	30.9 ± 3.3 ^b^	–1.7 ^†^	33.2 ± 3.5	32.2 ± 4.0 ^c^	–1.0 ^†^	–0.7 ^†^
	WC (cm)	94.9 ± 6.2	91.5 ± 7.8 ^c^	–3.4 ^†^	98.5 ± 11.2	96.9 ± 11.4	–1.6 ^†^	–1.8 ^†^
	BF (%)	44.3 ± 4.9	41.8 ± 6.1 ^c^	–2.5 ^†^	45.6 ± 3.5	44.0 ± 3.9 ^b^	–1.6 ^†^	–0.9 ^†^
	SBP (mm Hg)	121.4 ± 5.6	110.3 ± 8.5 ^b^	–11.1 ^†^	120.0 ± 8.8	114.4 ± 6.8 ^c^	–5.6 ^†^	–5.5 ^†^
	DBP (mm Hg)	77.2 ± 6.0	69.2 ± 6.2 ^b^	–10.0 ^†^	75.6 ± 7.7	72.4 ± 6.6	–3.2 ^†^	–6.8 ^†^
	TG (mmol/L)	0.89 ± 0.27	0.90 ± 0.32	0.01 ^†^	0.90 ± 0.35	1.00 ± 0.43	0.1 ^†^	–0.09 ^†^
	HDL (mmol/L)	1.29 ± 0.30	1.33 ± 0.27	0.04 ^†^	1.31 ± 0.28	1.30 ± 0.26	–0.01 ^†^	0.05 ^†^
	HOMA-IR	3.35 ± 2.49	2.51 ± 0.96	–0.84 ^†^	2.94 ± 1.31	2.93 ± 1.33	–0.01 ^†^	–0.83 ^†^
	ISI	4.60 ± 2.70	5.65 ± 2.36 ^c^	1.05 ^†^	3.98 ± 1.63	3.65 ± 1.40	–0.33 ^†^	1.38 ^†^
Ackel-D’Elia et al. [54]	BW (kg)	97.22 ± 13.06	89.12 ± 12.13 ^c^	–8.14 ± 4.57 *	96.11 ± 12.69	90.46 ± 10.81 ^c^	–5.14 ± 4.54	–3 ^†^
	BMI (kg/m^2^)	35.10 ± 4.67	31.82 ± 3.90 ^c^	–3.28 ± 1.56 *	35.06 ± 3.90	33.22 ± 3.70 ^c^	–1.85 ± 1.60	–1.43 ^†^
	BF (%)	45.57 ± 6.04	38.68 ± 6.27 ^c^	–6.80 ± 3.03 *	41.79 ± 6.59	41.07 ± 8.15	–1.18 ± 3.83	–5.62 ^†^
	FBG (µU/mL)	4.97 ± 0.39	4.96 ± 0.32	−0.01 ± 0.38	5.01 ± 0.34	4.97 ± 0.42	−0.05 ± 0.33	0.04 ^†^
	HOMA-IR	3.32 ± 1.17	2.54 ± 1.28	−0.78 ± 1.44	4.29 ± 2.75	3.97 ± 2.41	−0.32 ± 1.55	–0.46 ^†^
Bianchini et al. [55]	BW (kg)	82.4 (48.3–114.2)	81.1 (46.5–113.7)	−1.3 ^†^	82.3 (51.3–132.1)	83.6 (48.1–132.0) ^c^	1.3	−2.6^†^
	BMI (kg/m^2^)	31.7 (25.5–50.8)	30.8 (24.1–50.3) ^c^	−0.9 ^†^	30.6 (25.1–42.4)	30.4 (25.3–43.2)	−0.2 ^†^	−0.7 ^†^
	BF (%)	50.5 (23.5–58.4)	47.9 (16.5–56.7) ^c^	−2.6 ^†^	45.8 (24.1–56.0)	45.2 (24.0–55.4)	−0.6 ^†^	−2.0 ^†^
	BF (kg)	39.7 (14.6–64.6)	37.6 (10.0–61.6) ^c^	−2.1 ^†^	38.3 (13.4–56.6)	38.2 (14.4–60.1)	−0.1 ^†^	−2.0 ^†^
	WC (cm)	90.8 (74.5–122.0)	88.2 (70.8–125.0) ^c^	−2.6 ^†^	92.8 (73.0–116.0)	90.8 (74.5–112.0)	−2.0 ^†^	−0.6 ^†^
	SBP (mmHg)	121.0 (98.0–164.0)	118.0 (103.0–136.0) ^c^	−3.0 ^†^	124.5 (103.0–161.0)	122.5 (109.0–161.0)	−2.0 ^†^	−2.0 ^†^
	DBP (mmHg)	74.0 (57.0–100.0)	70.5 (57.0–86.0) ^c^	−3.5 ^†^	72.0 (54.0–126.0)	73.5 (54.0–104.0)	1.5 ^†^	−5.0 ^†^
	HDL (mg/dL)	45.5 (35.0–71.0)	44 (35.0–69.0)	−1.5 ^†^	44.5 (30.0–79.0)	43.0 (35.0–84.0)	−1.5 ^†^	−0 ^†^
	TG (mg/dL)	106.5 (51.0–209.0)	93.0 (40.0–383.0)	−13.5 ^†^	101.5 (40.0–280.0)	113.5 (44.0–286.0)	12 ^†^	−26.5 ^†^
	FBG (mg/dL)	88.0 (66.0–107.0)	87.0 (70.0–112.0)	−1.0 ^†^	89.0 (72.0–109.0)	84.5 (66.0–100.0) ^c^	−4.5 ^†^	3.5 ^†^
	HOMA-IR	3.7 (0.8–9.4)	3.2 (0.9–13.6)	−0.5 ^†^	3.7 (1.1–10.4)	3.8 (0.9–20.1)	0.1 ^†^	−0.6 ^†^
Toulabi et al. [56]	BW (kg)	81.67 ± 10.94	77.50 ± 12.46	–4.17 ^†^	84.43 ± 10.79	83.51 ± 11.79	–0.92 ^†^	–3.25 ^†^
	BMI (kg/m^2^)	30.43 ± 2.39	27.51 ± 3.18	–2.42 ^†^	30.33 ± 1.93	29.15 ± 2.74	–1.18 ^†^	–1.24 ^†^
	WC (cm)	99.25 ± 7.73	96.23 ± 9.50	–3.05 ^†^	100.19 ± 9.7	100.40 ± 6.99	0.21 ^†^	–3.26 ^†^
Peña et al. [57]	BW (kg)	86 ± 2	88 ± 2 ^b^	2	89 ± 4	92 ± 4 ^a^	3	–1.3
	BMI (z-score)	2.19 ± 0.04	2.15 ± 0.05	–0.04	2.28 ± 0.07	2.24 ± 0.09	–0.04	–0.009
	BMI (kg/m^2^)	32.1 ± 0.5	32.1 ± 0.6	0	33.7 ± 1.1	34.0 ± 1.2	0.3	–0.3
	BF (%)	44.5 ± 0.6	42.7 ± 0.7 ^b^	–1.8 ^†,^*	47.0 ± 0.9	46.3 ± 1.0	–0.7	–1.0
	ISI	1.8 ± 0.2	2.6 ± 0.4 ^b^	0.8	1.6 ± 0.2	2.8 ± 0.5 ^b^	1.2	–0.4
Peña et al. [58]	BW (kg)	90 ± 2	92 ± 2	2	95 ± 4	98 ± 4 ^a^	3	−0.8
	BMI (z-score)	2.25 ± 0.03	2.23 ± 0.04	−0.02	2.33 ± 0.10	2.32 ± 0.07	−0.01	−0.02
	BMI (kg/m^2^)	33 ± 1	34 ± 1	1	35 ± 1	35 ± 1 ^c^	0	−0.2
	WC (cm)	106 ± 1	107 ± 2	1	110 ± 3	111 ± 3	1	−0.8
	BF (%)	45 ± 1	44 ± 1	−1 *	47 ± 1	47 ± 1	0	−1.0
	ISI	1.9 ± 0.2	2.6 ± 0.3 ^a^	0.7	1.9 ± 0.3	2.5 ± 0.5	0.6	0.1
	FBG (mg/dL)	101 ± 1	99 ± 1 ^c^	−2	103 ± 1	106 ± 4	3	−4.7
Kitzman-Ulrich et al. [59]	BMI z score	NR	NR	0.0 ± 0.1	NR	NR	0.0 ± 0.1	0.0^†^
Reinehr et al. [60]	WC (cm)	103 ± 12	102 ± 13 ^c^	–1 ^†,^*	103 ± 11	107 ± 10 ^a^	4 ^†^	–5 ^†^
	FBG (mmol/L)	4.8 ± 0.4	4.9 ± 0.4	0.1 ^†^	4.8 ± 0.4	4.9 ± 0.4	0.1 ^†^	0 ^†^
	HDL (mmol/L)	1.3 ± 0.3	1.3 ± 0.4	0.0 ^†^	1.3 ± 0.3	1.3 ± 0.3	0.0 ^†^	0 ^†^
	TG (mmol/L)	1.3 ± 0.7	1.3 ± 0.7	0.0 ^†^	1.3 ± 0.7	1.3 ± 0.7	0.0 ^†^	0 ^†^
	SBP (mmHg)	127 ± 17	120 ± 15 ^a^	–7 ^†,^*	120 ± 14	122 ± 15	2 ^†^	−9 ^†^
	DBP (mmHg)	69 ± 12	67 ± 12 ^c^	–2 ^†,^*	64 ± 11	67 ± 12 ^b^	3 ^†^	−5 ^†^

*Notes:* BF: Body fat; BMI: Body mass index; BW: Body weight; CG: Control group; DBP: diastolic blood pressure; DiD, difference in differences (ΔIG–ΔCG); FBG: Fasting blood glucose; HDL: High-density lipoprotein; HOMA-IR: Homeostatic Model Assessment for Insulin Resistance; IG: Intervention group; ISI: Insulin sensitivity indices; NR: non-reported; SBP: Systolic blood pressure; TG: Triglycerides; WC: Waist circumference. The significance of pre-postintervention changes is indicated as follows: ^a^ *p* < 0.001, ^b^ *p* < 0.01, ^c^ *p* < 0.05. * *p* < 0.05 vs. ΔCG. All data reported in the table are from the studies included, except those marked with ^†^, which were calculated by the authors of this review from available data. Data are expressed as mean ± SD, except for (i) the two studies of Peña et al. expressed as mean ± SE; Bianchini et al. expressed as means (ranges).

**Table 3:** Results of the ROB 2 tool and the Jadad scale for each study considered.

Study	Domain 1. Randomization Process	Domain 2. Deviations from Intended Interventions	Domain 3. Missing Outcome Data	Domain 4. Measurement of the Outcome	Domain 5. Selection of the Reported Result	Domain 6. Overall Bias	Jadad Scale
Truby et al. (2016) [51]	Low risk	High risk	Low risk	Some concerns	Low risk	High risk	3
Plavsic et al. (2019) [53]	Some concerns	Low risk	Low risk	Some concerns	Low risk	Some concerns	3
Peña et al. (2023) [57]	Some concerns	Low risk	Low risk	Low risk	Some concerns	Some concerns	3
Peña et al. (2022) [58]	Low risk	Low risk	Low risk	Low risk	Low risk	Low risk	4
Ackel-D’Elia et al. (2013) [54]	Low risk	Low risk	Some concerns	Some concerns	Low risk	Some concerns	3
Toulabi et al. (2011) [56]	Low risk	Low risk	Some concerns	Some concerns	Low risk	Some concerns	3
Kitzman-Ulrich et al. (2009) [59]	Low risk	Some concerns	Low risk	Low risk	Some concerns	Some concerns	3

**Table 4:** Results of the ROBINS-I tool for each study considered.

Study	D1	D2	D3	D4	D5	D6	D7	Overall
Normayanti et al. (2020) [52]	Moderate	Low	Moderate	Low	Low	Moderate	Low	Moderate
Bianchini et al. (2012) [55]	Moderate	Low	Low	Low	Low	Moderate	Low	Moderate
Reinehr et al. (2009) [60]	Low	Low	Low	Moderate	Moderate	Low	Low	Moderate

D1, Bias due to confounding; D2, Bias in the selection of participants into the study; D3, Bias in the classification of interventions; D4, Bias due to deviations from intended interventions; D5, Bias due to missing data; D6, Bias in the measurement of outcomes; D7, Bias in the selection of the reported result.

## Data Availability

Data are contained within the article.
